# Letter to editor: Critical care beyond organ support: the importance of geriatric rehabilitation

**DOI:** 10.1186/s13613-024-01363-6

**Published:** 2024-08-19

**Authors:** Ken Hillman

**Affiliations:** 1 Simpson Centre for Health Services Research, South West Sydney Clinical School, University of New South Wales, Liverpool, NSW Australia; 2 School of Clinical Medicine, University of New South Wales, Liverpool, NSW Australia; 3 Ingham Institute for Applied Medial Research, Liverpool, NSW Australia

May I congratulate the authors on their contribution to this, one of the more important and pressing challenges in intensive care medicine - appropriate management of our increasingly aged population in intensive care [1]. The theme of the article is embodied in the following statement from the article: ‘The restoration of functional integrity and the associated impairment in overall quality of life are to be considered to be both the goal and main patient centred outcome measures in that age cohort’. The article then summarises the existing approach to rehabilitation for people aged > 80 years of age, including multidisciplinary teams embedded in the intensive care unit (ICU) to begin the rehabilitation process which would then continue in the post-hospital period.

The article assumes that the admission to the ICU is the main issue, and that the rehabilitation process is related to the ICU admission without considering the context. For example, common reasons for admission include falls and infection as a result of increasing frailty and decreasing reserves. Many in this cohort may be normally and naturally dying and are not recognised as such; that the admission to the ICU is simply part of the normal trajectory of people who are terminally ill (i.e. within the last 12 months of life). Over 60% of patients > 80 years of age who are admitted to an ICU are not alive at six months and a strong predictor of outcome was their pre-admission frailty level [2]. The fallback position of the elderly who are terminally ill is often hospitalisation and many of these spend their last few days in an ICU [2]. Over one third of all hospital emergency calls are for patients who are near the end of life and have not been recognised until they seriously deteriorate in the hospital and most of these are elderly [3].

The article is based on input from the specialty of geriatrics who have expertise in rehabilitation but who may not be aware of the terminally ill status of patients. The Geriatric Syndrome is a key document for the specialty of geriatrics which summarises the common complications associated with ageing and how to manage them [4]. Ironically, dying and death and how to manage these inevitable events is not mentioned in the document.

A medical profession focussed on curing and a society which is reluctant to discuss dying is a powerful complicity. Intensive care is the often the end of an inevitable conveyor belt taking terminally ill elderly patients from their homes into hospital and into ICUs. Ironically, many elderly people do not want to be admitted to hospitals and most do not want to die there. As well as being involved in rehabilitation, intensivists must familiarise themselves with the prognostic data for elderly people and how to recognise those in the last 12months of life. All health professionals need to be involved in empowering them to ensure their own goals of care are made clear and are respected by the health profession. While the emphasis in this article on restoration of functional integrity can be important, equally important is recognising the elderly nearing the end of life and delivering management consistent with their terminally ill status and above all, being honest with the patient and their carers.

## Data Availability

Not applicable.

## Declarations

### Authors’ information (optional)

If any of the sections are not relevant to your manuscript, please include the heading and write ‘Not applicable’ for that section – Not applicable.
